# Effect of Cellulose–Chitosan Hybrid-Based Encapsulation on the Viability and Stability of Probiotics under Simulated Gastric Transit and in Kefir

**DOI:** 10.3390/biomimetics7030109

**Published:** 2022-08-10

**Authors:** Muhammad Afzaal, Farhan Saeed, Huda Ateeq, Yasir Abbas Shah, Muzzamal Hussain, Ahsan Javed, Ali Ikram, Muhammad Ahtisham Raza, Gulzar Ahmad Nayik, Saleh Alfarraj, Mohammad Javed Ansari, Ioannis K. Karabagias

**Affiliations:** 1 Department of Food Sciences, Government College University Faisalabad, Faisalabad 38000, Pakistan; 2 Department of Food Science and Biotechnology, Graduate School, Kyungpook National University, Daegu 41566, Korea; 3 Department of Food Science & Technology, Government Degree College Shopian, J&K 192303, India; 4 Zoology Department, College of Science, King Saud University, Riyadh 11451, Saudi Arabia; 5 Department of Botany, Hindu College Moradabad, Mahatma Jyotiba Phule Rohilkhand University, Bareilly 244001, India; 6 Department of Food Science & Technology, School of Agricultural Sciences, University of Patras, G. Seferi 2, 30100 Agrinio, Greece

**Keywords:** encapsulation, emulsion, polysaccharides, kefir, simulated gastrointestinal conditions

## Abstract

Encapsulation comprises a promising potential for the targeted delivery of entrapped sensitive agents into the food system. A unique combination of cellulose/chitosan (Cl-Ch)-based hybrid wall material was employed to encapsulate *L. plantarum* by emulsion technique. The developed beads were further subjected to morphological and in vitro studies. The viability of free and encapsulated probiotics was also evaluated in kefir during storage. The developed beads presented porous spherical structures with a rough surface. A 1.58 ± 0.02 log CFU/mL, 1.26 ± 0.01 log CFU/mL, and 1.82 ± 0.01 log CFU/mL reduction were noticed for Cl-Ch hybrid cells under simulated gastro-intestinal and thermal conditions, respectively. The encapsulated cells were found to be acidic and thermally resistant compared to the free cells. Similarly, encapsulated probiotics showed better viability in kefir at the end of the storage period compared to free cells. In short, the newly developed Cl-Ch hybrid-based encapsulation has a promising potential for the targeted delivery of probiotics, as career agents, in gastric transit, and in foods.

## 1. Introduction

There is currently consumer demand for functional foods that provide health benefits beyond adequate nutrition and potentially prevent diseases. This is a growing trend, driven by the increased awareness of health, quality of life, and rising health costs. Probiotic-based products are key players in the functional food market due to their association with gastrointestinal health [1]. Probiotics induce a positive effect on the host’s health if consumed in an appropriate quantity (10^6^–10^7^ CFU/g). These are living cultures of microorganisms that improve the overall well-being of their user [2]. In view of their beneficial role in their host’s health, these have been in high demand over the last decade (FAO/WHO Working Group, 2002). The potential benefits of probiotics include anti-mutagenic and anti-carcinogenic, host’s immune system stimulation/activation, blood cholesterol level reduction, lactose intolerance management, and the general improvement in nutritional status [3,4].

Probiotics cannot withstand harsh conditions, i.e., low pH, oxidizing agents, and antimicrobial agents in food formulations. Sensitivity to high acidity in the stomach and less tolerance to bile salts and bile acids in the small intestine are the key factors in the log reduction in viable probiotics cell count. Furthermore, throughout the storage period of food products, maintaining probiotics’ cell viability has always been a matter of great concern for the food processing industry. Currently, research is underway to enhance their stability during the processing and storage of probiotics-supplemented products until their consumption. It is further expected that these probiotics, after ingestion, must retain their viability in the low pH environment of the gastrointestinal (GI) tract and deliver their benefits by colonizing the gut [5].

Few strains of bacteria, such as *Lactobacillus reuteri,* contain high tolerance toward low pH conditions [6], yet the majority of the bacteria cannot tolerate pH at 2; therefore, it can be deduced that pH is the major regulator of microbial diversity [7]. The protective coatings being researched and developed for drug delivery are not usually in line with the viability and growth of probiotics and other microorganisms. In the light of recent studies, it is recommended that one hundred million (10^8^) viable probiotic bacteria must colonize the gut to obtain health benefits [8]. Kefir is a fermented product that originated many years ago in the Caucasian mountains of Tibet or Magnolia. Kefir is mainly produced from sheep milk, but in Europe, it is produced on a small scale from cow milk. In the development of innovative functional foods, microencapsulation is currently considered the most effective strategy to protect probiotics against hostile conditions during the processing and storage of foods. There are a variety of microencapsulation techniques available, but the most popular and effective are chilling, emulsion, extrusion, and spray-drying [9].

It is vital that encapsulation does not decrease the viability and activity of the probiotic by providing a shield against harsh conditions during processing, storage, and in the GI tract. The material most suited to be used as membrane material for encapsulation should be easy to handle, biocompatible, non-toxic, easily available, and costless [10]. Different wall materials can be utilized as encapsulating materials to provide protection to probiotics. Cellulose is an exclusively used plant-based material [11]. Other types of cellulose, i.e., carboxymethyl cellulose, can also be used with other polymers to form complexes [12]; therefore, chitosan and cellulose-based coatings materials are extensively being used in probiotics encapsulation [13]. Chitosan is obtained by the deacetylation of chitin and contains randomly organized α(1→4) 2-amino 2-deoxy β-D-glucan units. Kefir granules are not regular in size gelatinous masses, and range in size from 1–6 mm in diameter [14]. Considering the aforementioned, the aim of the present study was to investigate the effect of cellulose–chitosan hybrid encapsulated cells on the viability of *Lactobacillus plantarum* underexposed simulated digestive fluids and in kefir.

## 2. Materials and Methods

Encapsulating materials, chemicals, and glassware were purchased from Merck (USA). The freeze-dried culture of *Lactobacillus plantarum *(ATTCC 8826) and Kefir grains were obtained from the National Institute of Food Science and Technology (NIFSAT), University of Agriculture Faisalabad, Pakistan. Raw milk was purchased from the local farm of Faisalabad (Pakistan) and stored at 4 °C.

### 2.1. Activation of Bacterial Culture 

Activation of the freeze-dried cell was performed by the following method by Afzaal et al. [15], with some modifications. Cells of *L. plantarum* (ATTCC 8826) were inoculated anaerobically in 100 mL of DeMan, Rogosa, and Sharpe (MRS) broth for propagation. The bacterial culture was incubated at 37 °C for 24–48 h. Afterwards, the obtained cells were centrifuged (750286 EA, Thermo Fisher Scientific Inc. Waltham, MA, USA) 3000 rpm for 15 min, and the media was decanted. Cells were again suspended in freshly prepared MRS media and incubation was carried out at 37 °C for 20 h. The cells were harvested, and the concentration was adjusted to 10^10^ CFU/mL. 

### 2.2. Preparation of Cellulose and Chitosan-Based (Cl-Ch) Capsules

By adopting the procedure of Li et al. [16], with slight modifications, cellulose-based encapsulated beads were prepared. The combination of 4.6% *w/v* alkaline solution and 5% *w/w* cellulose was mixed at 25 °C for 14 h to make a cellulose solution. To remove air bubbles from the solution, it was centrifuged at 8000 rpm for 8–10 min at 4 °C. Afterwards, the cellulose solution (45 mL) was poured and well-mixed with bacterial culture (2 g) and 1 g Tween 80 solution along with 180 mL paraffin oil. Then, the solution was stirred using a magnetic stirrer (200 rpm) at 20 °C. By continuous stirring for about 2.5 h, diluted HCl (1.5% *w/v*) was poured in order to maintain a neutral pH. CaCl_2_ was added dropwise (2–3 mL) to harden the prepared beads and afterwards, the obtained cellulose-based microbeads were washed gently with distilled water and a small amount of hot ethanol at 45 °C was also added for the removal of residual paraffin and oil traces. Finally, clean and dry microbeads were obtained at the end of the process. 

Probiotics were coated in a chitosan layer by using a 1% *w/v* solution of chitosan, according to Santos et al. [17], with slight modifications. For the process, 2.5% *w/w* chitosan was added to distilled water to make a 100 mL solution. Afterwards, the liquid solution was acidified by the addition of 0.1M glacial acetic acid (0.5–1 mL *w/v*) and the pH of the solution was dropped to 3.5. Then, the solution was autoclaved at 121 °C for 15 min just to make the solution aseptic before utilization of the solution. The solution was cooled down at ambient temperature and probiotic culture (2 g) was added. The whole solution was then stirred with the help of a magnetic capsule at 500 rpm for about 25–30 min. CaCl2 was added dropwise (2–3 mL) in order to obtain mature beads; as a result, beads were formed, and were then washed with distilled water and stored at 4 °C for further use. 

For the preparation of Cl-Ch hybrid beads, the cellulose solution 5% *w/v* was poured and well-mixed using an alkaline solution. Meanwhile, 3% chitosan solution (*w/v*) was mixed in solution at a 2:1 ratio and the whole solution was heated to 87 °C. The solution was cooled down at room temperature and bacterial culture (2 g), along with 180 mL paraffin oil. Afterward, the solution was stirred at 200 rpm at 20 °C continuously for about 30 min. The Cl-Ch-based hybrid microbeads obtained were hardened using CaCl_2_ solution (1–2 mL) that was added dropwise to the solution. Mature beads were obtained by this process and were washed gently with distilled water and some amount of ethanol (8–10 mL at 40 °C) was added just for the removal of residual paraffin and oil traces. Finally, clean and dry microbeads were obtained at the end of the process.

### 2.3. Characterization of Beads

For the examination of beads, a light microscope (DSX11000 Model, Olympus Technologies Ltd., Huddersfield, England.) was used. As previously reported by Yıldız-Akgül et al. [18], the size and diameter of the prepared beads were evaluated. 

#### Zeta Potential Measurement

The Zeta potential of the prepared beads was measured by following the method of Ding et al. [19]. The prepared beads were dispersed in distilled water and zeta potential was measured using a Zeta potentiometer (Zetasizer ZSU1002, Malvern, Worcestershire, UK) at 30 Kv. The solution was kept for a few minutes to settle, and the zeta potential was measured.

### 2.4. Encapsulation Efficiency

The encapsulation efficiency or entrapment efficiency was calculated by adopting the method of Yasmin et al. [20]. The mean results for the viability of *L. plantarum* in both Cl-Ch hybrid beads, cellulose, and chitosan beads were obtained by disintegrating the beads separately in a 9 mL (*w/v*) sterile solution of sodium citrate having a neutral pH. After the release of live cells in the solution, the enumeration of cells was carried out by following the serial dilution procedure. For this purpose, peptone water (0.1% *w/v*) was used following the pour plate method. The Petri dishes were incubated for 48 h at 37 °C (Incubator IN55plus, Schwabach, Germany. After the incubation process, probiotic growth was counted by using a manual laboratory colony counter (model SC6Plus, LabX, Chelmsford, England). The encapsulation efficiency was estimated using the following formula:

$$\mathrm{Encapsulation}\;\mathrm{Yield}\%=\mathrm{EY}\%=\left(\frac{\mathrm{Log}10N}{\mathrm{Log}10N0}\right)\times \;100$$

where N = The number of viable entrapped cells that are released from the beads, N0 = The number of free cells added prior to encapsulation.

### 2.5. SEM

A scanning electron microscope of high resolution (Cube series-. Emcraft, Gwangju-si, Korea), available at the Physics Department-GCUF, was used. The microbeads were subjected to structural characterization as described by De Wever et al. [21] with slight modifications. The beads were analyzed using 10.00 kV power.

### 2.6. Release Study of Encapsulated Beads in Simulated Gastric Fluid

The survival of free (unencapsulated) and encapsulated cells was evaluated by adopting the process described by Zhang et al. [22] with minor modifications. Briefly, simulated gastric fluid (SGF) was obtained by the dissolution of 2.5–3 g/L pepsin in sterile saline 1% (*v/v*) and the pH was adjusted to 2.0 by adding diluted HCl (1M). The prepared solution was heated to 37 °C in an incubator, encapsulated and un-encapsulated cells were then added in separate tubes (0.5 g) for 120 min. All tubes were shaken to mix the cells and kept again in an incubator at 37 °C under anaerobic conditions with constant shaking at 800 rpm. After every 30 min, the viable cells were determined with a maximum time of 120 min.

### 2.7. Release Study of Encapsulated Beads in Simulated Intestinal Fluid

For the study of the viability of encapsulated and free cells of *L. plantarum* in SIF, the procedure of Xu et al. [23] was followed with minor modifications. The solution was prepared following the protocol and warmed in an incubator before cell inoculation. Encapsulated and free cells (0.5 g) were added in separate tubes of sterile SIF solution for 120 min. The free cells (0.5 mL) and encapsulated cells (0.5 g) were separately added to tubes containing 4.5 mL of sterile SIF. All tubes were shaken to mix the cells and kept again in an incubator at 37 °C under anaerobic conditions with constant shaking at 800 rpm. After every 30 min, the viable cells were determined. 

### 2.8. Thermal Resistance

The thermal stability of encapsulated probiotics and unencapsulated probiotics was evaluated according to the method reported by Sabikhi et al. [24], with slight modifications. Briefly, 1 g of free and encapsulated cells were inoculated in the tubes that contained sterile distilled water (10 mL) and subjected to various temperatures for 10 min at 50 °C, 60 °C, 70 °C, and 80 °C. The viable count (log CFU/g) was calculated after thorough vortexing of the samples for a few minutes, then followed by MRS agar plating and enumeration after 72 h at 37 °C.

### 2.9. Product Development (Kefir)

Kefir was produced by the method of Sanchez et al. [25], with some slight modifications. For the preparation of Kefir, raw cow milk (purchased from the local farm in Faisalabad) was pasteurized at 65 °C for 30 min and allowed to cool down at ambient temperature. Afterward, inoculation of kefir grains was performed (2% *w/w*) and these were kept under 25 °C for fermentation for 12–15 h, so that the pH reached the value of 4.5. Afterward, the whole mixture was divided into five portions: control (without any probiotic addition), free probiotics, and encapsulated probiotics (encapsulated with cellulose, chitosan, and cellulose–chitosan). The latter were added at the level of 10^9^ CFU/mL and the curd was stirred. All the kefir samples were kept at 4 °C for 15 days. 

#### 2.9.1. Characterization of Kefir

The pH of Kefir was determined using a digital pH meter (Mettler Toledo121, Greifensee, Switzerland). 

#### 2.9.2. Probiotic Enumeration

For the selective enumeration of *L. plantarum*, the method of Yıldız-Akgül et al. [18] was followed with some modifications. Serial dilutions were prepared for the inoculation of Kefir samples in order to determine probiotic enumeration. The sample solution was spread on the Petri dishes containing MRS agar for the growth of probiotic bacteria. All the plates were kept in an incubator at 37 °C for 46–72 h. Small circular colonies were obtained having a diameter of 2–3 mm and colonies were enumerated using a colony counter. 

### 2.10. Statistical Analysis

The experiments were planned with a fully randomized design and analyzed by means of a one-way analysis of variance (ANOVA). ANOVA was carried out with Statix 10 (Tallahassee, FL, USA ), and multiple comparisons were used for data analysis and for comparing the average values at the confidence level *p* < 0.05. All the experiments were conducted in triplicate (*n* = 3). 

## 3. Results and Discussion 

### 3.1. Bead Size and Shape

Beads’ characteristics are a key factor in encapsulation technique; however, the size and composition depend on the type of polymers used. All beads obtained were spherical in shape and exhibited whitish-yellow color, which is due to the natural color of the coating materials used in the study. The size of Cl-Ch-based microbeads was 3.56 ± 1.12 mm. Moreover, the structure of Cl-Ch beads was less porous as compared to the cellulose and chitosan beads and the pores were not of uniform size. Hence, these pores are not large enough to release the encapsulated probiotics in the surrounding fluids, therefore, providing a better barrier to probiotics in comparison to cellulose and chitosan beads. In the study of Li et al. [26], it was also reported a porous spherical cell structure of the cellulose-based polymer was obtained during the encapsulation. In this study, the size of chitosan-coated beads was 2.79 ± 0.16 mm. It should be noted that the type of coating material used may have an impact on the size of beads, as suggested by Valero-Cases and Frutos [27].

### 3.2. Zeta Potential of Encapsulated Beads

Zeta potential (ζ-potential) is an important parameter that defines electrochemical properties and surfaces electrical attributes of free and encapsulated microbeads. The mean significant results for the zeta potential of microbeads having cellulose and chitosan wall materials are shown in Table 1. The results indicated that the type of encapsulating materials greatly affects the zeta potential of beads. The zeta potential of *L. plantarum* encapsulated with Cl-Ch hybrid wall material was −26 ± 0.07 mV. The increase in the zeta potential may be attributed to the inherent negative charge of gel under neutral conditions; therefore, with the increase in the concentration of the wall material, the stability of microcapsules also increases.

### 3.3. Encapsulation Yield

The results for encapsulation efficiency showed a high and significant (*p* < 0.05) value for cellulose-based cells; however, chitosan provided a lower acceptable yield% of probiotics (78% ± 1.02) and cellulose-based capsules showed an efficiency of 80% ± 2.41. The best results were obtained for cellulose–chitosan-based probiotics (87% ± 1.82). The reason for such high bacterial yield is that cellulose forms a layer around probiotics that protect them and, in turn, protect the cell membranes from any kind of damage and disruption. *L. plantarum* was previously encapsulated by Chotiko et al. [28] using rice bran and they obtained a high bacterial yield in the range of 80–95%. They further concluded that a high yield can be obtained by using a high concentration of wall materials. 

### 3.4. Scanning Electron Microscopy (SEM)

**Figure 1:**
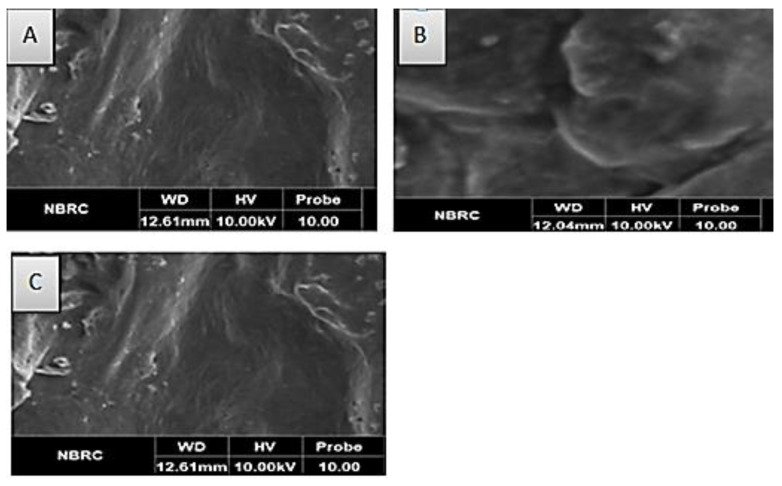
Scanning micrographs of encapsulated probiotics. (**A**) C_ch_ (*L. plantarum* encapsulated with chitosan). (**B**) C_cl_ (*L. plantarum* encapsulated with cellulose). (**C**) C_ch-cl_ (*L. plantarum* encapsulated with chitosan and cellulose hybrid cells).

The SEM fingerprint for each of the encapsulated probiotics with *L. plantarum* is shown in Figure 1A–C. As shown in Figure 1A–C, pores were observed in all types of encapsulated cells. Additionally, all pores were not homogenous. The presence of a dark surface was also observed in the beads. The presence of the probiotics in coated materials showed that the used wall materials are important in augmenting the viability and stability of probiotics under hostile conditions.

### 3.5. Release Study of Encapsulated Beads in Simulated Gastric Fluid

**Figure 2:**
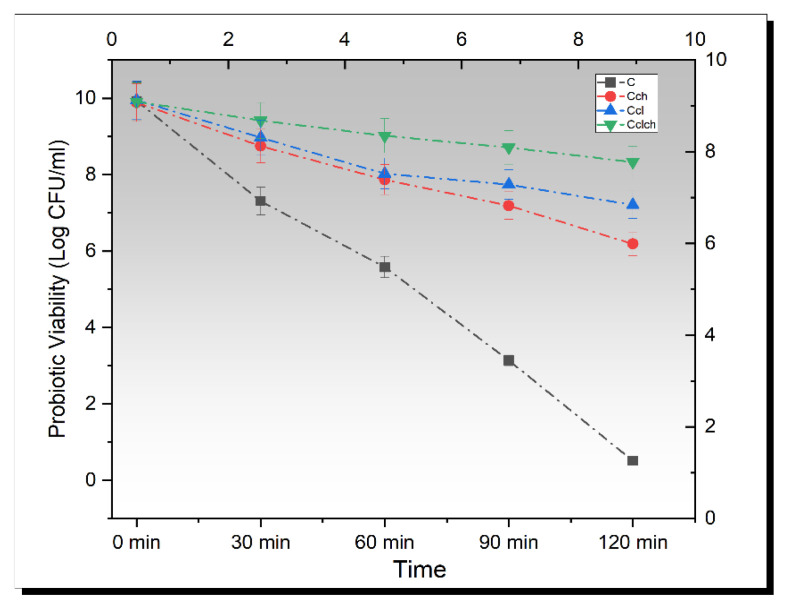
Viability of un-encapsulated and encapsulated (cellulose, chitosan, and cellulose–chitosan hybrid cells) probiotic microgels under simulated gastric fluid conditions during storage intervals (0, 30, 60, 90, and 120 min) compared with control. Each line represents mean value for viability of treatments. C (un-encapsulated probiotics), Cch (*L. plantarum* encapsulated with chitosan), Ccl (*L. plantarum* encapsulated with cellulose), and Cch-cl (*L. plantarum* encapsulated with chitosan and cellulose hybrid cells).

By exposing the probiotic cells to simulated gastric fluid, a more precise estimate of resistance to upper gastrointestinal transit can be observed. The stomach environment can be detrimental for probiotics since it is a part of the digestive tract. Apart from pepsins and salts, the major characteristic of the gastric solution is its lower pH. As a result, it can be challenging for probiotics to be able to withstand such environments with lower pH [29]. The initial cell population was set in the range of 9.5–10 log CFU and the beads were inoculated in a simulated gastric solution. The mean results for all cell types (free/encapsulated) regarding simulated gastric environments are shown in Figure 2. All the cells showed a significant (*p* < 0.05) decreasing trend. The free cells of *L. plantarum* showed a rapid downfall in population, which showed that the free probiotic cells cannot survive in the low acidic environments of the stomach; however, the maximum population was observed in cellulose–chitosan-coated probiotic cells. The reason for this finding might be that the probiotic cells entered the center of the cellulose microgels, given that the cellulose microgels have a permeable structure that allowed them to fit in [30]; however, further research is still required in this field. Moreover, a log reduction of 9.42 ± 0.01 CFU/mL and 1.58 ± 0.02 CFU/mL was observed in free *L. plantarum* and cellulose–chitosan hybrid cells. 

### 3.6. Release Study of Encapsulated Beads in Simulated Intestinal Fluid

Live probiotic cells can never survive in the low pH conditions of the human gut, and hence, cannot retain their activity [31]. Krasaekoopt and Bhandari [32] suggested that free probiotics, especially LAB, can no longer maintain their survival at stomach pH, but if these are covered by microcapsules, they can resist in such an acidic environment even after 2 h of incubation.

**Figure 3:**
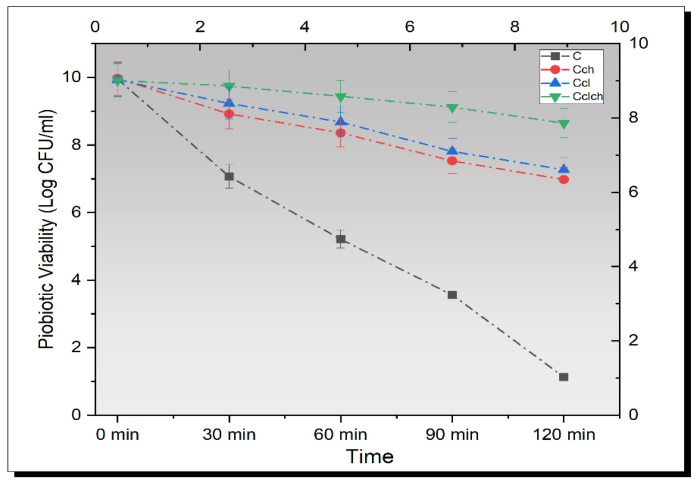
Viability of un-encapsulated and encapsulated (cellulose, chitosan, and cellulose–chitosan hybrid cells) probiotic microgels under simulated intestinal fluid during storage intervals (0, 30, 60, 90, and 120 min) compared with control. Each line represents mean value for viability of treatments. C (un-encapsulated probiotics), Cch (*L. plantarum* encapsulated with chitosan), Ccl (*L. plantarum* encapsulated with cellulose), and Cch-cl (*L. plantarum* encapsulated with chitosan and cellulose hybrid cells).

In order to improve the viability of probiotics in the gut, one important parameter is their resistance to the hard acidic environment of the stomach and intestine. The initial cell population was set in the range of 9.5–10 log CFU and the beads were inoculated in a simulated gastric solution. The mean results from a triplicate set of experiments of all treatments are shown in Figure 3. A significant (*p* < 0.05) decreasing trend was observed from the graph of bacterial survivability under simulated intestinal fluid. The maximum value for viable probiotics was obtained from Cl-Ch-coated probiotics, while Ch-coated cells showed lower resistance toward intestinal fluid. A log reduction of 1.26 ± 0.01 CFU/mL was calculated for Cl-Ch hybrid microbeads, while a log reduction of 2.99 ± 0.01 CFU/mL and 2.66 ± 0.01 CFU/mL was noted for chitosan and cellulose-based microcapsules, respectively; however, free probiotics showed a sharp fall in the bacterial population after inoculation. The reason behind this was that the probiotic cells entered the center of the cellulose microgels. The hybrid microgels have a porous structure, whereas the chitosan and cellulose beads have non-uniform pores that are comparatively larger than hybrid cells, which makes the hybrid cells retain more probiotics and do not provide space for their release in the surrounding environment. Hence, hybrid beads take a longer time to resolve in the solution, whereas the cellulose microgels and chitosan microgels have a more porous structure that allows them to dissolve in the solution and release the bacteria uncovered in the solution [30]. 

In another study for hybrid carboxymethyl cellulose/*k*-carrageenan polymers for the encapsulation of *L. plantarum*, a high, survival rate after exposure to bile condition was observed, and hybrid capsules displayed effective protection against the damage of the bile salt solution. This phenomenon can be explained by the fact that a double-layer blend offers lower porosity and a thicker structure, which can result in preventing bile entrance through the blend network [33].

### 3.7. Probiotic Enumeration in Kefir

**Figure 4:**
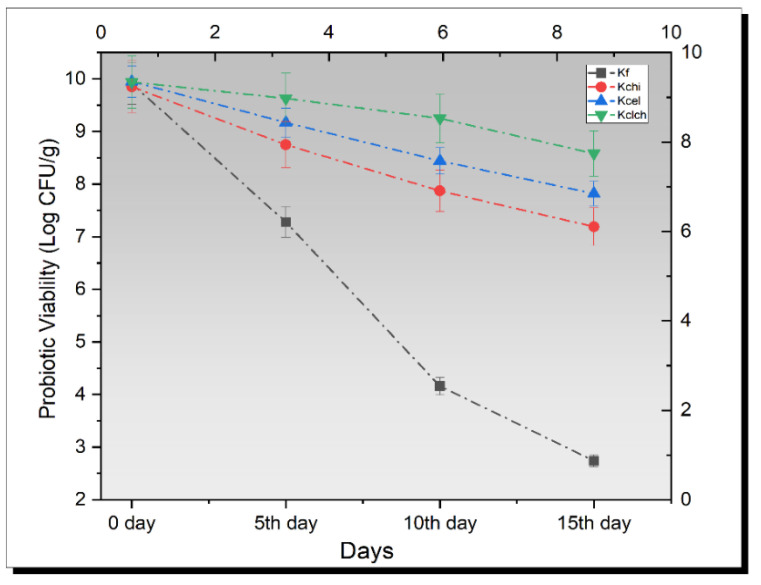
Viability of un-encapsulated and encapsulated (cellulose, chitosan, and cellulose–chitosan hybrid cells) probiotic microgels on the probiotic viability in kefir during storage interval (0, 5th, 10th, and 15th day) compared with control. Each line represents mean value for viability of treatments. C (un-encapsulated probiotics), Cch (*L. plantarum* encapsulated with chitosan), Ccl *(L. plantarum* encapsulated with cellulose), and Cch-cl (*L. plantarum* encapsulated with chitosan and cellulose hybrid cells).

Mean results for probiotic viability are shown in Figure 4 Overall, a gradual significant decreasing trend was observed. The maximum probiotic viability was observed in kefir that was inoculated with Cl-Ch hybrid cells. A log reduction of 1.36 ± 0.01 CFU/g was observed in kefir loaded with Cl-Ch hybrid cells, while a log reduction of 2.13 ± 0.01 CFU/g was determined in kefir having cellulose-coated probiotics. In the case of kefir with chitosan encapsulated cells, a log reduction of 2.66 ± 0.03 CFU/g was monitored. The maximum value for log reduction was observed in kefir with free probiotics (7.17 ± 0.02 log CFU/g). Similar research for the growth of probiotic bacteria was conducted by Dinkçi et al. [34], and they found a relatively higher probiotic count. In addition, a relevant research study was also carried out by Güzel-Seydim et al. [35], and the authors suggested similar probiotic enumeration as in the present study. 

Yilmaz et al. also conducted a study on kefir that revealed a decrease in the viable count of approximately 0.5 log for the survival of alginate-starch encapsulated cultures of *L. acidophilus* and Bifidobacterium spp; however, probiotic viability of *L. paracasei* KS-199 in kefir could be increased by encapsulation within biopolymers and biopolymer plays an essential role for encapsulation of probiotics with increased viability in kefir.

### 3.8. Thermal Resistance

**Figure 5:**
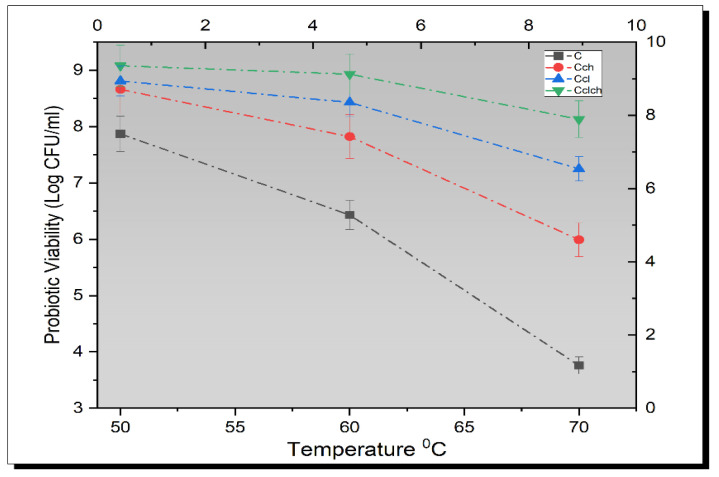
Effect of free (unencapsulated) and encapsulated (chitosan and cellulose) *L. plantarum* on probiotic viability in Kefir at elevated temperatures (50 °C, 60 °C, and 70 °C) compared with control. Each line represents mean value for viability of treatments. Kf (un-encapsulated probiotics/free), Kch (Kefir containing *L. plantarum* encapsulated with chitosan), Kcl (Kefir containing *L. plantarum* encapsulated with cellulose), and Kch-cl (Kefir containing *L. plantarum* with cellulose–chitosan hybrid cells).

Previous studies have suggested that some gram-positive bacteria, including species of genera Lactobacillus, are heat resistant and can survive up to 75 °C; therefore, to prove this fact, bacterial species were subjected to heat shock (50 °C, 60 °C, 70 °C, and 80 °C) for 10 min. The obtained results showed a significant (*p* < 0.05) decrease in the bacterial population with an increase in temperature (Figure 5). Although the maximum peak was obtained for Cl-Ch-coated bacterial cells, the results from free probiotics showed a sharp decline in the viability under elevated heat shock, as shown in Figure 4. This is due to the fact that higher temperature causes the probiotic structure to unfold and denature the protein and other amino acid components, thus leading to the death of the live probiotic cells [36]. Lian et al. [37] suggested that the wall materials that are used for encapsulation have different physical properties and can act as a barrier against several adverse conditions. 

## 4. Conclusions

Probiotic survival in carrier foods or functional foods developed with a certain hypothesis is essential for extracting the associated health benefits. In this context, encapsulation is an effective approach for maintaining the viability and stability of probiotics under detrimental conditions. Cellulose and chitosan-based wall materials were evaluated for the protection of probiotics under stressed conditions, separately and in combination. A significant effect was noted between encapsulated and non-encapsulated cells in terms of survival under different hostile conditions. Encapsulated probiotics also showed better viability and stability in a carrier food (kefir) during storage. Cellulose–chitosan hybrid beads are effective wall materials in the delivery of sensitive ingredients such as probiotics. Additionally, these wall coating materials are suitable for maintaining the therapeutic number of probiotics in a carrier or a developed functional food during storage. 

## Figures and Tables

**Table 1:** Size, encapsulation efficiency, and zeta potential of encapsulated cells.

Cell Type	Size (mm)	Encapsulation Efficiency (%)	Zeta Potential
Cch	2.79 ± 0.16	78 ± 1.02	−32 ± 0.03 mV
Ccl	3.18 ± 0.15	80 ± 2.41	−15 ± 0.09 mV
Cch-cl	3.56 ± 1.12	87 ± 1.82	−26 ± 0.07 mV

The data are shown as average ± SD during the analysis of three replicates (*n* = 3).

## Data Availability

Even though adequate data have been given in the form of tables and figures, all authors declare that if more data are required, then data will be provided on request.
